# Effect of Specific Mutations in Cd300 Complexes Formation; Potential Implication of Cd300f in Multiple Sclerosis

**DOI:** 10.1038/s41598-017-12881-8

**Published:** 2017-10-19

**Authors:** Águeda Martínez-Barriocanal, Andrea Arcas-García, Miriam Magallon-Lorenz, Aroa Ejarque-Ortíz, María Luciana Negro-Demontel, Emma Comas-Casellas, Simo Schwartz, Sunny Malhotra, Xavier Montalban, Hugo Peluffo, Margarita Martín, Manuel Comabella, Joan Sayós

**Affiliations:** 1 CIBBIM-Nanomedicine-Immune Regulation and Immunotherapy Group. Institut de Recerca Vall Hebrón (VHIR), Universitat Autònoma de Barcelona, Barcelona, Spain; 2 CIBBIM-Nanomedicine-Drug Delivery and Targeting Group. Institut de Recerca Vall Hebrón (VHIR), Universitat Autònoma de Barcelona, Barcelona, Spain; 3 Servei de Neurologia-Neuroimmunologia. Centre d’Esclerosi Múltiple de Catalunya (Cemcat). Institut de Recerca Vall Hebrón (VHIR), Universitat Autònoma de Barcelona, Barcelona, Spain; 4 Networking Research Center on Bioengineering, Biomaterials and Nanomedicine (CIBBER-BBN), Instituto de Salud Carlos III, Barcelona, Spain; 5 Biochemistry Unit, Institut d’Investigacions Biomèdiques August Pi i Sunyer, Faculty of Medicine, University of Barcelona, Barcelona, Spain; 6 Neuroinflammation and Gene Therapy Laboratory, Institut Pasteur Montevideo, Montevideo, Uruguay; 7 Department of Histology and Embryology, Faculty of Medicine, UDELAR, Montevideo, Uruguay

## Abstract

Herein, we have used bioinformatics tools to predict five clusters defining ligand-binding sites on the extracellular domain of human CD300b receptor, presumably involved in the formation of both homodimers and heterodimers with other CD300 family members. Site-directed mutagenesis revealed residues glutamic acid 28 and glutamine 29 in cluster 5 to be necessary for the formation of CD300b complexes. Surprisingly, the disruption of cluster 2 and 4 reconstituted the binding capability lost by the mutation of residues glutamic acid 28 to alanine, glutamine 29 to alanine (E28A-Q29G). We identified a missense mutation arginine 33 to glutamine (R33Q) in CD300f by direct sequencing of exon 2 in peripheral blood samples from 50 patients with multiple sclerosis (MS). Levels of expression of CD300f were almost undetectable on monocytes from the patient bearing the R33Q mutation compared with healthy individuals. Whereas R33Q mutation had no effect in the formation of CD300f complexes, the inhibition of protein synthesis with cycloheximide indicated that CD300f R33Q is less stable than native CD300f. Finally, we report that the levels of expression of CD300f on the surface of classical and intermediate monocytes from MS patients are significantly lower when compared to the same cell populations in healthy individuals.

## Introduction

Immune function is the result of integrating positive and negative signals delivered by activating and inhibitory receptors, predominantly on the surface of leukocytes. These receptors are responsible for the transduction of information from the surrounding environment to the interior of the cell and launch a response according to the nature and intensity of the stimulus. Human CD300 immune receptors are type I transmembrane proteins expressed mainly on the surface of cells of the myeloid linage^1^. The seven members of the human CD300 family are clustered in chromosome region 17q25.1 and encode both inhibitory and activating receptors. CD300b, CD300c, CD300e and CD300h trigger activating signals through different molecular mechanisms^2–5^, whereas CD300a and CD300f behave as inhibitory receptors by recruiting SHP-1 phosphatase^6,7^. It is worth mentioning that CD300f presents a functional duality, as it has been also shown to deliver activating signals through the recruitment of PI 3-kinase and $\mathrm{Fc}{\rm{\varepsilon }}R$γ^2,8^. Finally, CD300d lacks intrinsic signaling capacity but displays regulatory features, as it restricts the surface expression of other CD300 receptors^9^.

Regarding CD300 ligand recognition, in recent years it has been reported that diverse phospholipids and sphingolipids act as ligands of various members of the human and murine CD300 family^10^. While it has been described that human CD300a and CD300c are capable of recognizing phosphatidylserine and phosphatidylethanolamine^11,12^, CD300f binds to sphingomyelin and ceramide^13^. So far, the exact nature of the ligands for the rest of the human CD300 receptors remains elusive. Similarly, diverse members of the murine CD300 family are described to bind both phospho- and sphingolipids^14–17^. The data obtained with diverse murine models have shown that the function of these molecules seems to be related to diverse cellular processes including efferocytosis, immune regulation and cytokine signaling. Moreover, although there are no data to date correlating human CD300 dysfunction with any pathological condition, the functional blockade of different CD300 receptors in mice has demonstrated their involvement in several pathological models, including autoimmune disorders^17,18^, MS^19^, nerve regeneration^20^, asthma^21^, colitis^22^, and acute kidney^23^ and brain damage^24^.

Our laboratory identified the capability of CD300 receptors to associate between them extracellularly, forming both homo- and hetero-signaling complexes^4^. A key fact is that the integration of CD300 molecules in complexes modifies the signaling properties of individual receptors allowing synergies at the same time as agonistic and/or antagonistic processes. In this report, we aimed to determine which residues are involved in the establishment of CD300 complexes. During the process, we have analyzed the existence of mutations in CD300f in MS patients and demonstrated that the levels of expression of CD300 expression on the surface of monocytes from MS patients are significantly lower than on monocytes from healthy individuals.

## Results

### Predicting ligand-binding sites on the extracellular domain of CD300b

**Figure 1:**
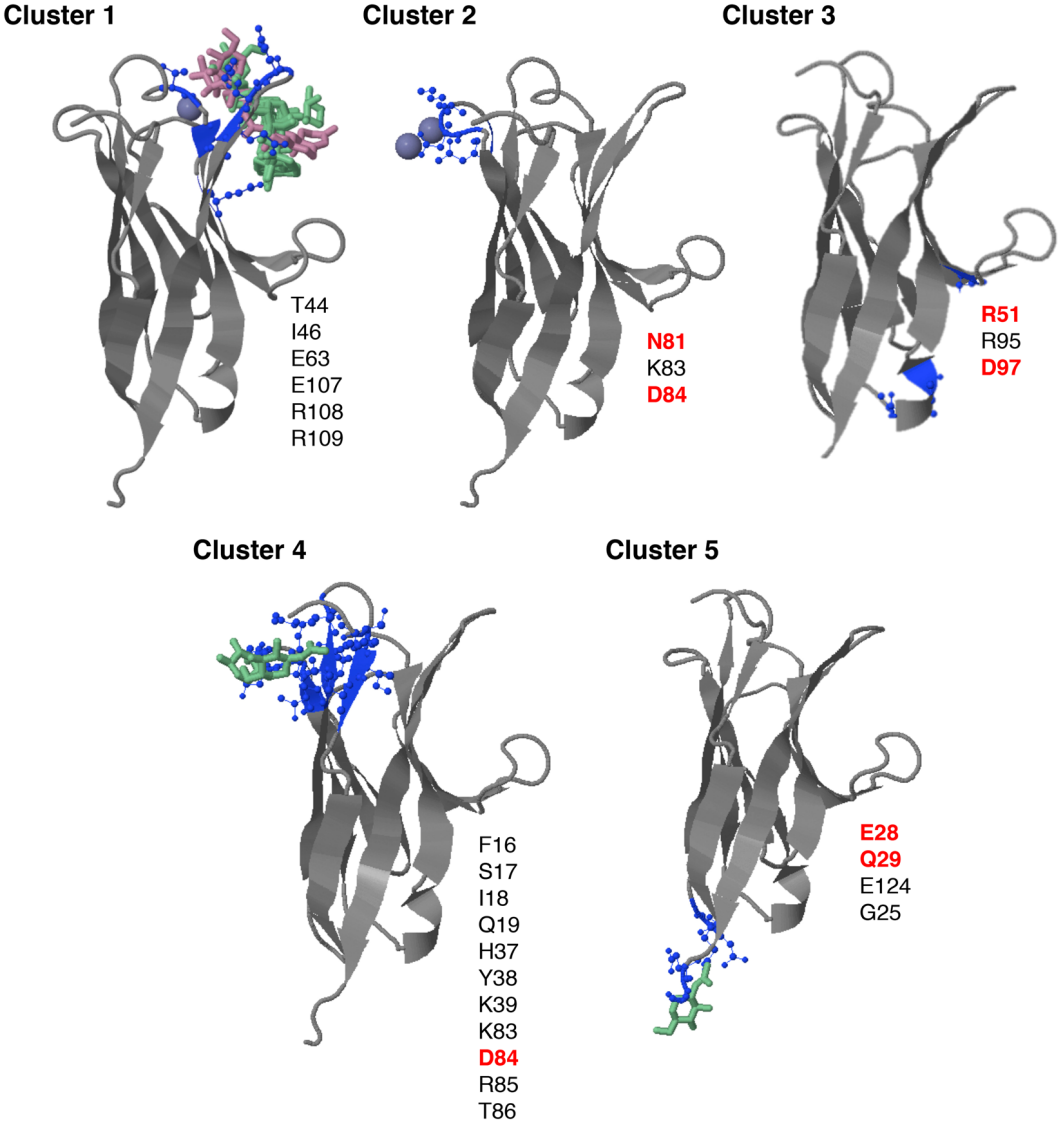
Prediction of ligand-binding sites on the CD300b extracellular domain. Schematic representation of the five predicted binding sites (3DLigandSite) within the extracellular domain of human CD300b. The amino acids defining each cluster are listed and shown in blue within the structure. Heterogens (glycan chains and ions) used to build the models are also shown. The amino acids highlighted in red were subjected to mutagenesis to assess their role in complex establishment.

CD300 receptors associate extracellularly forming both homo- and hetero-signaling complexes; however, the residues on the Ig domain leading to these interactions are not known. Due to the limited information regarding CD300b receptor crystal structure, we decided to address this question by means of bioinformatic prediction tools. We submitted the human CD300b immunoglobulin (Ig) domain sequence to the 3DLigandSite web server (http://www.sbg.bio.ic.ac.uk/3dligandsite). The server predicts first a protein structure using Protein Homology/analogY Recognition Engine (Phyre)^25^, which is then used to identify homologous structures with bound ligands within a library. Ligands are superimposed onto the modelled structure and clustered to define binding sites. We chose the CD300b extracellular domain first, because it is the simpler Ig domain among all of the CD300 receptors in terms of post-translational modifications, which are known not to be involved in CD300 complex formation but could influence the prediction server^4^. In contrast to other CD300 receptors, CD300b is not N-glycosylated and presents fewer O-glycosylation sites^3^. Second, CD300b is the receptor within the family in which homo- and hetero-complexes have been studied in depth, allowing us to rule out certain residues if predicted based on previous studies^4^. The results obtained by modeling the CD300b Ig domain revealed the existence of 5 different and well-defined clusters, as depicted in Fig. 1. Based on the previous data generated by our group^4^, aiming to determine which residues were involved in the CD300b complex formation, we initially discarded clusters 1, 2, and 4. Thus, we decided to focus our attention on clusters 3 and 5, which were defined by residues arginine 51, arginine 95, asparagine 97, and glutamic acid 28, glutamine 29, glutamic acid 124, glycine 125, respectively (Fig. 1).

### Mutagenesis of residues defining CD300b binding clusters 3 and 5

**Figure 2:**
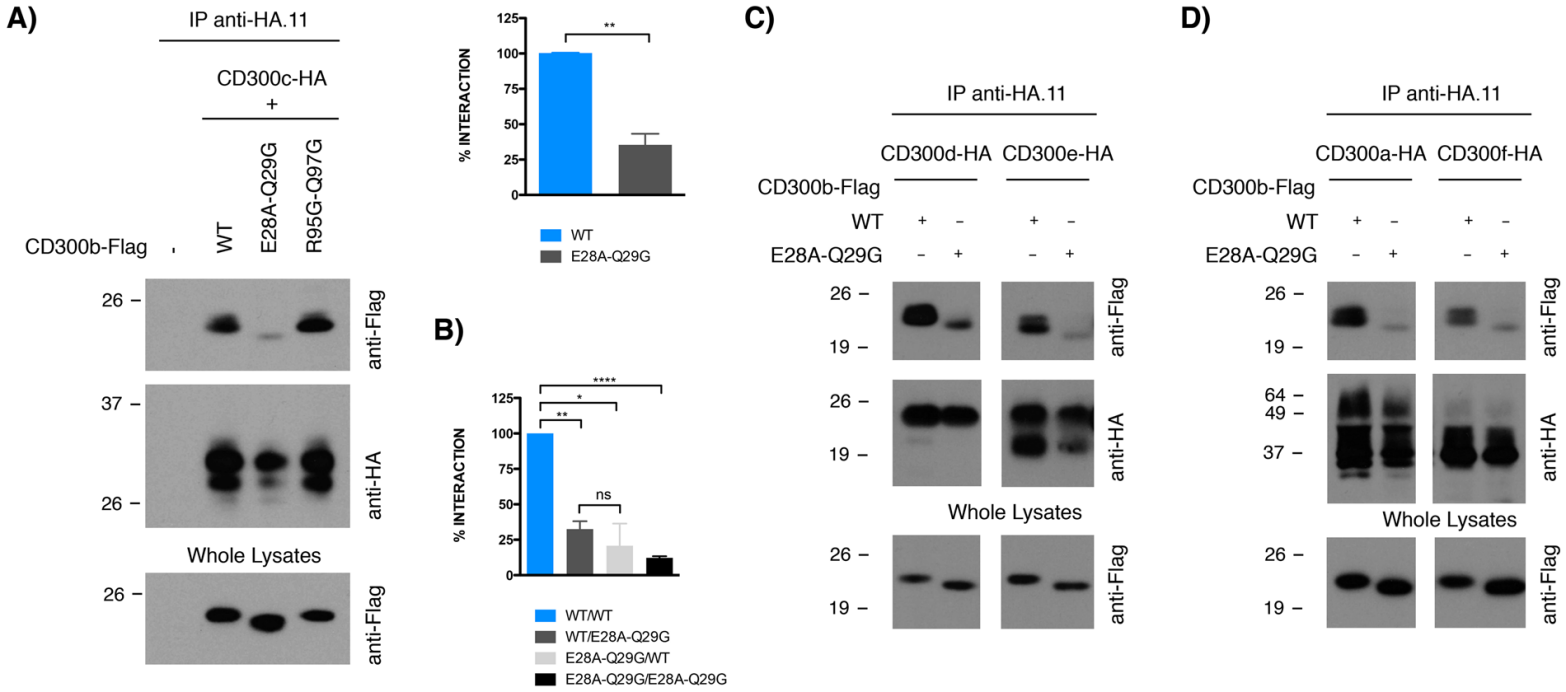
Analysis of the effect of disruption of clusters 3 and 5 in CD300b complexes formation. COS-7 cells were transiently co-transfected with a combination of HA- and Flag-tagged CD300 constructs. Cell were lysed and immunoprecipitated with anti-HA.11 mAb. Blots were probed with the indicated antibodies in order to determine the capability of receptors to form complexes. Whole cell lysates (2%) were included as loading and transfection controls. (**A**) HA-CD300b wt was tested against Flag-CD300b _R95G-Q97G_ and Flag CD300b _E28A-Q29G_. (**B**) HA- and Flag-tagged CD300b wt were tested against HA- and Flag-tagged CD300b _E28A-Q29G._ HA-CD300e and HA CD300d (**C**) and HA-CD300a and HA-CD300f (**D**) were tested against Flag-CD300b _E28A-Q29G_. Descriptive data were expressed as the arithmetic mean ± SD. The GraphPad Prism statistical package was used to investigate group differences by unpaired, two-tail Student’s t test. p values are indicated for statistically different means: * ≤ 0.05, ** ≤ 0.01, **** ≤ 0.0001. n.s: non-significant.

To test whether clusters 3 and/or 5 were participating in the interaction between the Ig domains of CD300 receptors, we generated CD300b point mutation constructs to perform co-immunoprecipitation experiments against CD300c, an extensively studied heterocomplex. Precisely, in order to disrupt cluster 3, we generated the double mutant CD300b_R95G-Q97G_, and similarly generated the mutant CD300b_E28A-Q29G_ to analyze the role of cluster 5. COS-7 cells, which lack endogenous CD300 expression, were co-transfected with HA-tagged CD300c and Flag-tagged CD300b in the wild type (wt) or mutant forms. It is worth mentioning that cell surface expression of both CD300b mutants was equivalent to the wt receptor (data not shown). Lysates from transfected cells were subjected to immunoprecipitation with an antibody against the HA epitope. Co-precipitated Flag-CD300b molecules were visualized by western blotting using an anti-Flag antibody. The CD300b_R95G-Q97G_ mutant bound as efficiently as the wt form to CD300c (Fig. 2A), whereas CD300b_E28A-Q29G_ binding was markedly reduced (Fig. 2A). Quantitatively, the CD300b_E28A-Q29G_ mutant bound 4-fold less efficiently to CD300c than the wt receptor. To analyze whether the disruption of cluster 5 was able to block the interaction of CD300b with itself, we co-transfected COS-7 cells with HA- and Flag-tagged CD300b wt and CD300b_E28A-Q29G_ constructs in all possible combinations (Fig. 2B). Interestingly, whereas the interaction between the wt and the E28A-Q29G mutant was notably impaired (4-fold reduction), the interaction between two mutant receptors was almost abolished. CD300b glutamic acid 28 and glutamine 29 residues were not only important for homodimerization and CD300c binding, but were essential for binding to all CD300 family members as well. The CD300b_E28A-Q29G_ mutant bound less efficiently to CD300e/CD300d (Fig. 2C) and CD300a/CD300f (Fig. 2D) when compared to the wt form.

### Functional effect of CD300b complex disruption

**Figure 3:**
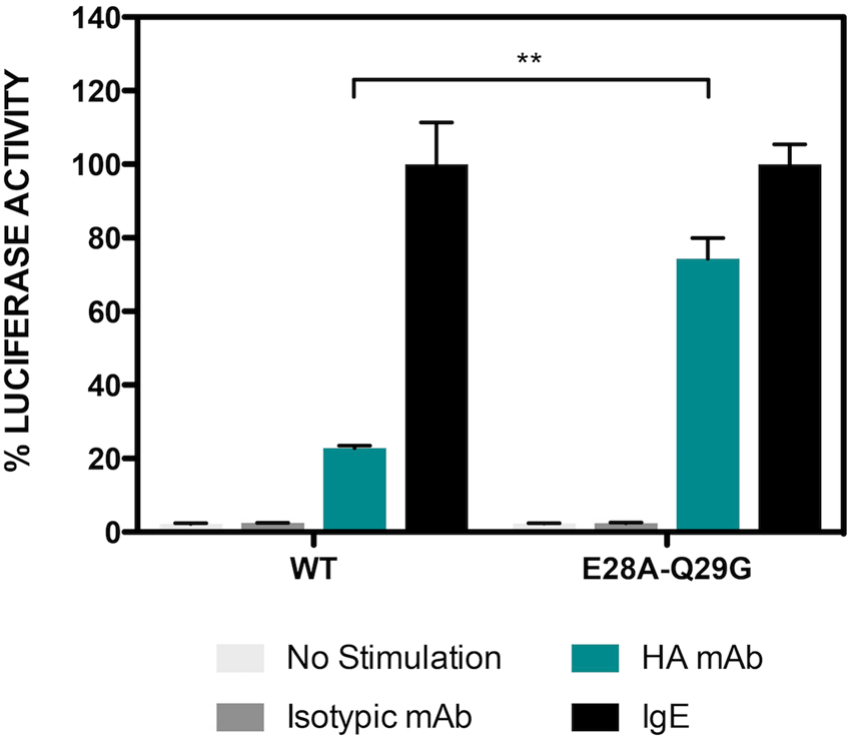
Blocking the formation of CD300b complexes enhances receptor signaling. (**A**) RBL-2H3 cells were stably transfected with HA-CD300b wt or HA-CD300b _E28A-Q29G_. RBL-2H3 transfectants were transiently transfected with 3 × NFAT/AP1-Luciferase and TK-Renilla reporter plasmids. Luciferase activity was measured after stimulation for 7 h with the indicated antibodies. Data were normalized and expressed as a percentage of luciferase activity considering IgE stimulation as the top threshold of activation. Duplicates were performed for all stimulations. The result is a mean ± SD of three independent experiments. Group differences were investigated by unpaired, two-tail Student’s t test. p values are indicated for statistically different means: ** ≤ 0.01.

We have previously shown that the composition of CD300 receptors within a heterocomplex determines the signaling outcome, representing a new mechanism by which CD300 complexes finely regulate the activation of myeloid cells upon interaction with their natural ligands^4^. In order to explore whether glutamic acid 28 and glutamine 29 residues, key for CD300b homocomplex formation, could regulate the functional properties of this receptor, we performed transcriptional activity assays in the RBL-2H3 cell line. This cell line, from myeloid origin, was first stably transfected with HA-tagged CD300b wt or CD300b_E28-Q29_ mutant and then transiently transfected with a luciferase reporter gene under the control of an NFAT/AP-1-dependent promoter. This reporter is activated by multiple activating immune receptors including CD300b or FcɛRI, the receptor for IgE^4^. The stimulation of cells with anti-HA mAb, mimicking the CD300b natural ligand, led to increased NFAT-AP1 transcriptional activity, which was further enhanced in the CD300b_E28A-Q29G_ mutant (Fig. 3). It is noteworthy that the overall activating capacity and cell surface levels of FcɛRI were conserved between transfectants (≥99% expression, data not shown).

### Disruption of clusters 2 and 4 reconstitutes the binding capability lost by mutation of residues glutamic acid 28 and glutamine 29

**Figure 4:**
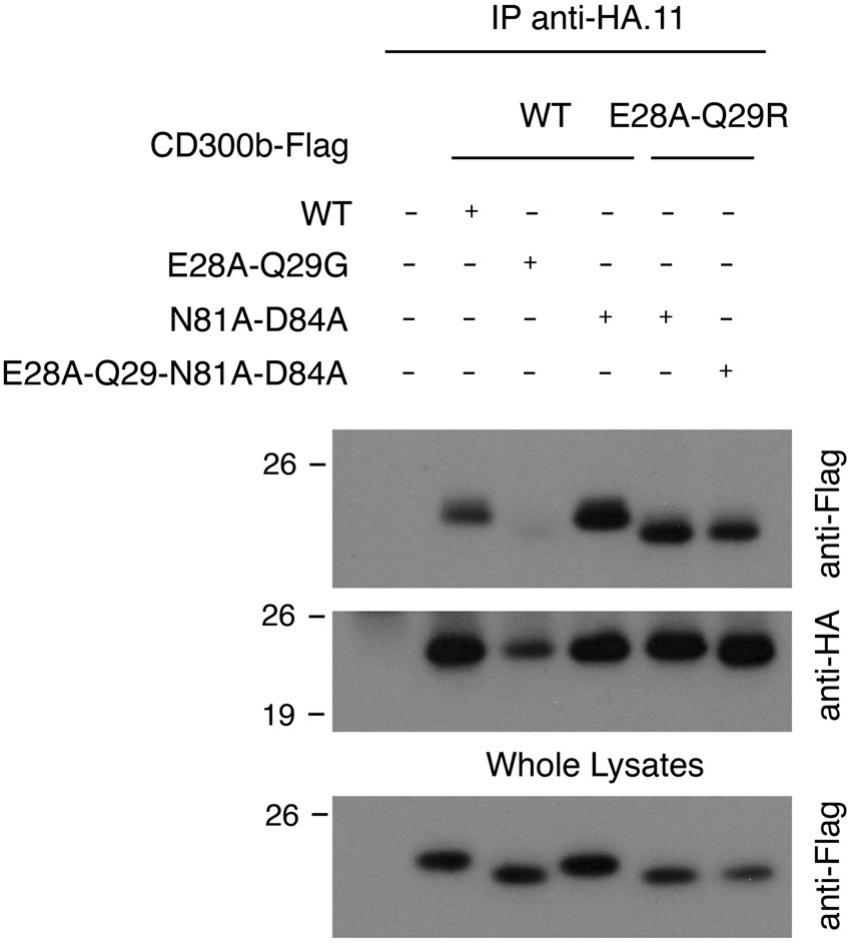
Disruption of cluster 2 reconstitutes the binding capability lost by disruption of cluster 3. COS-7 cells were transiently transfected with HA-CD300b wt in combination with Flag-tagged CD300b wt, Flag-CD300b_E28A-Q29G_, Flag-CD300b_N81A-D84A_ and Flag-CD300b_E28A-Q29G-N81A-D84A._ Cells were lysed and immunoprecipitated with anti-HA.11 mAb. Blots were probed with the indicated antibodies. Whole cell lysates (2%) were included as controls.

The data generated involved the residues on cluster 3 in the binding between CD300 Ig domains; however, it was conceivable that other residues on the Ig domain could also be participating in the binding. Even then, the effect observed was the result of variations in the tridimensional structure of CD300b Ig, such as changes in the orientation of the amino acids lateral chains within the beta-sheets, surface polarity or distance to the ligand, which precluded the complex formation. In order to test the last hypothesis, we focused our interest on clusters 2 and 4, which are defined around some common residues. We mutated amino acids asparagine 81 and aspartic acid 84, generating the mutant CD300b_N81A-D84A_. Again, the cell surface expression of CD300b mutant receptor in COS-7 cells did not differ from the native receptor (data not shown). The substitution of both residues by alanine had no effect on CD300b homocomplex formation (Fig. 4). Surprisingly, the mutant CD300b_N81A-D84A_ co-precipitated with the CD300b_E28A-Q29G_ construct. Moreover, even the quadruple mutant CD300b_E28A-Q29G-N81A-D84A_ was able to form complexes with CD300b_E28A-Q29G_ (Fig. 4). These data strongly suggest that none of the mutated residues are directly involved in interactions between CD300b Ig domains, but rather participate in establishment of the tridimensional structure of CD300b Ig, which is suitable for complex formation.

### SNPs on the Ig domain of CD300 receptors

**Figure 5:**
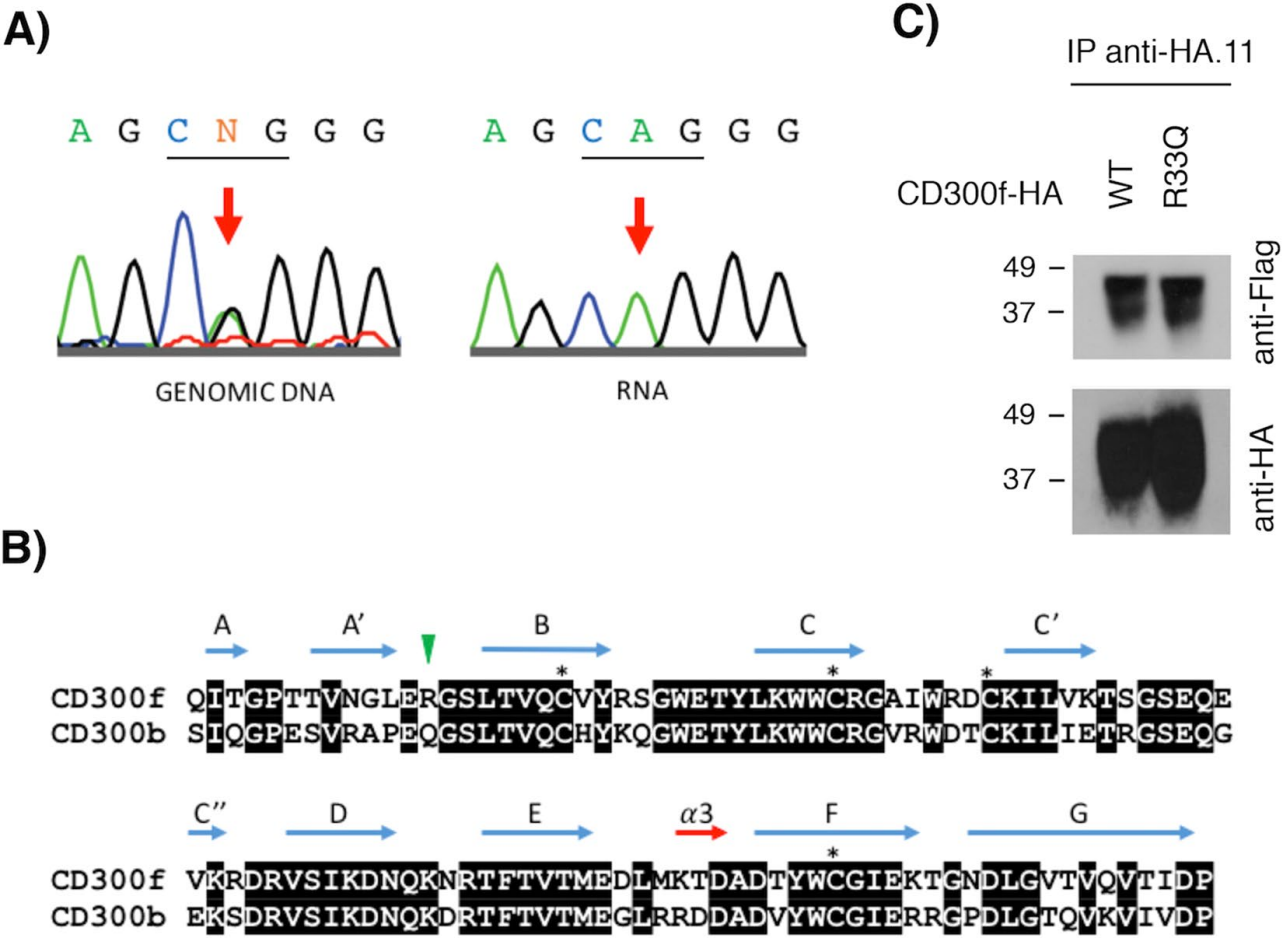
SNP identification in CD300f receptor. (**A**) Sequence chromatograms showing the genomic DNA (left) and vector-cloned cDNA (right) CD300f sequence covering SNP rs141171369 (17:72700901 C/T) identified in LF6105 patient. CD300f codon 33 has been underlined. (**B**) Protein sequence alignment of human CD300b and CD300f Ig-like domains. Identical residues are shown on black background. Cysteine residues involved in the Ig-like domain fold are identified with an asterisk. Secondary structure elements are shown above amino acid sequences. Residues Gln29 in CD300b and Arg33 in CD300f are highlighted with a green arrow. (**C**) COS-7 cells were transiently transfected with HA-CD300f wt in combination with Flag-CD300f wt or Flag-CD300f_R33Q._ Cells were lysed and immunoprecipitated with anti-HA.11 mAb. Blots were probed with the indicated antibodies.

Our previous data indicate that mutations and/or single nucleotide polymorphisms (SNPs) affecting the formation of CD300 complexes are able to modify the function of receptors. Despite there being no data correlating the dysfunction of human CD300b with any human disease, there are numerous reports showing that murine CD300f and CD300b play a similar role in the clearance of apoptotic cells, acting as scavenger receptors in autoimmune-related pathologies^16,17^. In fact, human CD300b and CD300f immunoglobulin domains present the highest homology among CD300 family of receptors. In the case of CD300f, some reports related the lack of this molecule with autoimmune diseases such as lupus and MS^18,19^. Considering that experimental autoimmune encephalomyelitis (EAE), the most commonly used experimental model for human MS, is exacerbated in CD300f KO mice^19^, we decided to explore the CD300 mutational landscape in human disease. Precisely, we sequenced exon 2 from human CD300b and CD300f genes, which encodes the whole Ig domain in both receptors, in fifty patients suffering from MS. Out of the 100 exons sequenced (two for each sample), we found one SNP (rs17553512) in the CD300b gene that affects nucleotide position 484 (G/A) and results in a synonymous substitution (isoleucine 120 to isoleucine). The frequency of both alleles (A 58–G 42%) was similar to the described for the European population in the NCBI SNP database (www.ncbi.nih.gov). In the case of CD300f, we identified two different SNPs in samples from MS patients. The first was a missense SNP (rs35489971) affecting nucleotide 22 (valine 22 to alanine). The distribution of both alleles (G 77–A 23%) was close to that described for the European population (G 82–A 18%). The second SNP (rs141171369), identified in a single patient, produces the missense mutation R33Q (Fig. 5A) and it was described in European, South American and African populations with a very low frequency (0.0002556) (http://exac.broadinstitute.org/variant/17-72700901-C-T). The alignment of CD300b and CD300f Ig domains (Fig. 5C) demonstrates that glutamic acid 32 and arginine 33 in CD300f resemble residues glutamic acid 28 and glutamine 29 in CD300b, whose mutation was found to abolish the formation of CD300b complexes. To fully confirm the presence of this SNP, we sequenced cloned cDNA retrotranscribed from RNA derived from isolated monocytes (Fig. 5A). In order to determine whether this SNP could somehow have an effect on the ability of CD300f to form complexes, we generated a HA-tagged CD300f_R33Q_ construct and co-transfected it in COS-7 cells with the Flag-tagged CD300f wt construct. As shown in Fig. 5C, the substitution of arginine 33 by a glutamine residue did not affect the formation of CD300f homocomplexes. In fact, even the substitution of both glutamic acid 32 and arginine 33 by alanine and glycine, respectively, did not impair the capability of CD300f to form complexes (data not shown).

### Functional effect of SNP rs141171369 on CD300f expression

**Figure 6:**
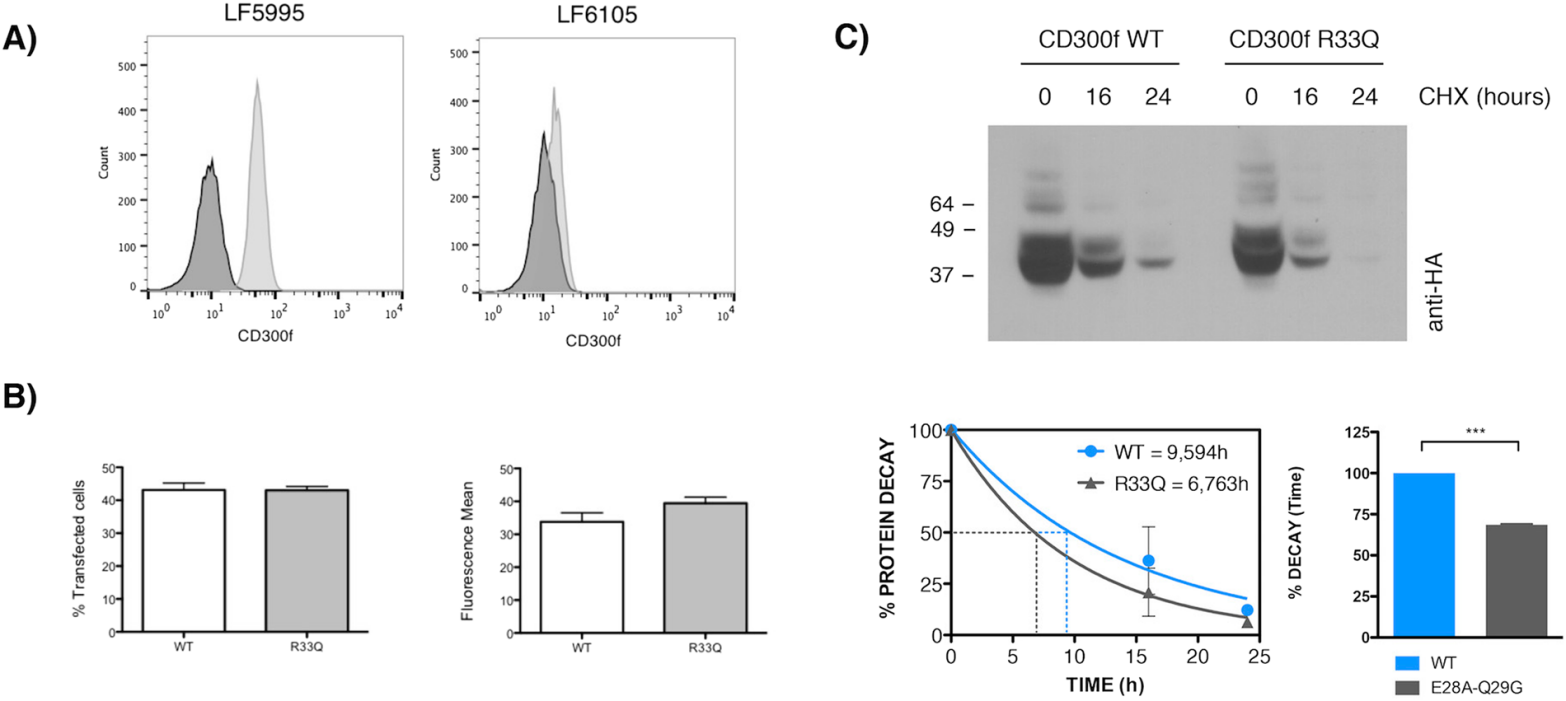
Functional consequences of SNP rs141171369 in CD300f. (**A**) Monocytes from a healthy individual (LF5995, right) and the MS patient (LF6105) were stained with anti-CD300f monoclonal antibody (UPD2) (light gray histogram) and an isotypic mAb as a negative control (dark gray histogram). (**B**) COS-7 cells were transiently transfected with Flag-CD300f wt or Flag-CD300f_R33Q_ and CD300f cell surface expression was monitored 48 hours post-transfection as described above (% of CD300f positive cells and mean fluorescence intensity are shown) or (**C**) protein synthesis was inhibited by adding cycloheximide to the cell culture for the indicated time-points. Cells were lysed and cell lysates were subjected to western blot as described. Receptor half live was calculated using GraphPad Prism (phase decay non-linear regression) and differences were investigated by unpaired, two-tail Student’s t test. p values are indicated for statistically different means: *** ≤ 0.001.

In order to determine whether the heterozygous substitution R33Q in CD300f could affect the function of the receptor, we first analyzed by flow cytometry the level of expression of CD300f on the surface of peripheral blood circulating monocytes from the MS patient in which the substitution was identified. As shown in Fig. 6A, whereas CD300f was on the cell surface of 100% of healthy control monocytes, only 6% of monocytes from patient LF6105 expressed CD300f. To determine whether the R33Q mutation was responsible for the low levels of CD300f surface expression detected on monocytes, COS7 cells were transfected with wt and R33Q mutant CD300f constructs. As shown in Fig. 6B, the expression levels of CD300f wt and R33Q mutant on the surface of COS-7 transfected cells were similar. Next, we analyzed whether the substitution of arginine 33 could modify the stability of CD300f and thus, decrease its half-life. Cycloheximide is a classical inhibitor of protein biosynthesis known by its ability to interfere with the translocation step in protein synthesis. By treating transfected COS-7 cells with cycloheximide in a time-course experiment followed by Western blotting against CD300f, we observed that CD300f_R33Q_ was less stable than CD300f wt (Fig. 6C). The blockade of protein synthesis for 24 h hours reduced the amount of wt CD300f protein, whilst the levels of CD300f_R33Q_ were almost undetectable under the same experimental conditions.

### Expression of CD300f on monocytes from MS patients

**Figure 7:**
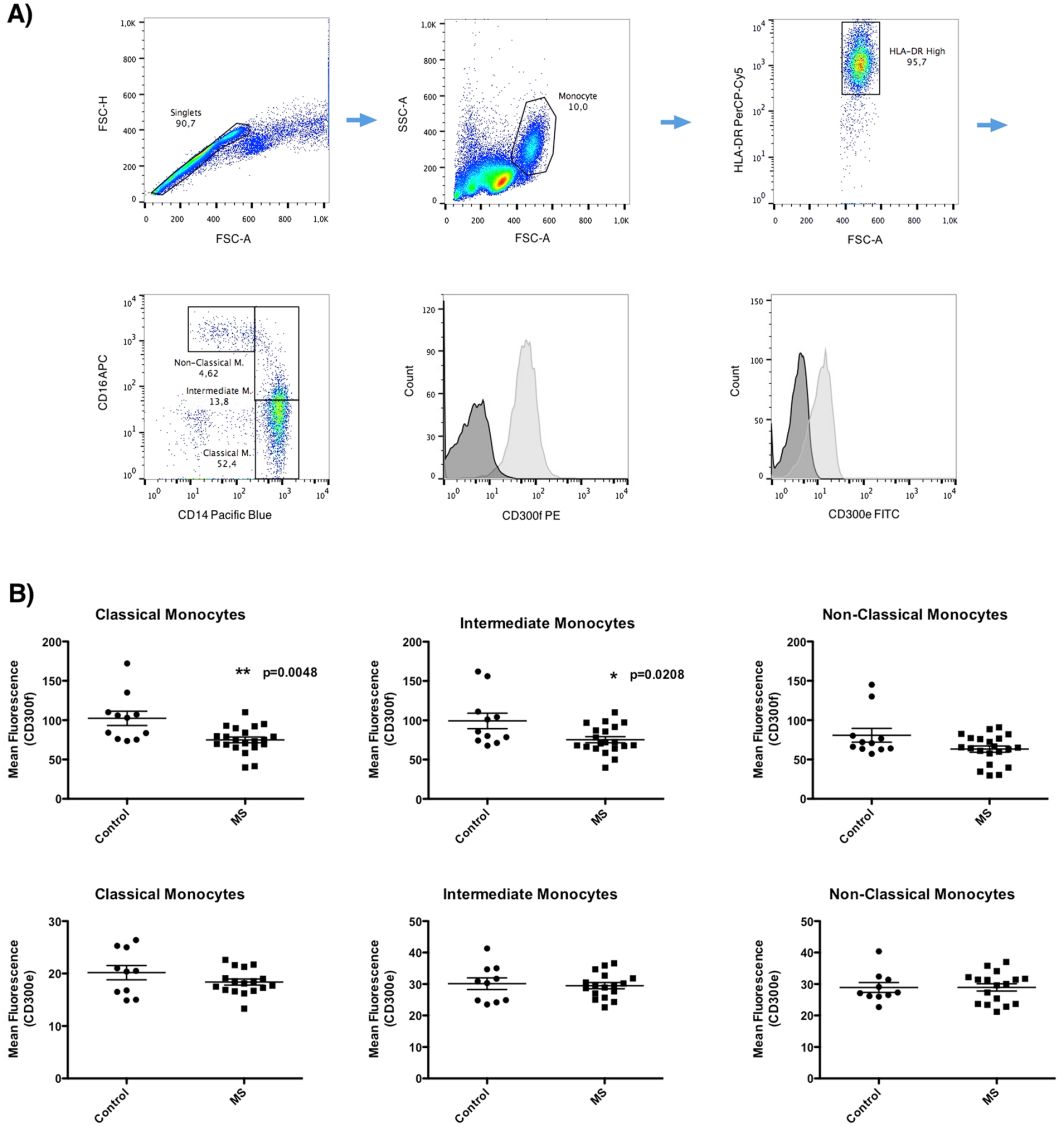
Expression of CD300f on MS patient’s monocytes. (**A**) Gating strategy to define the three populations of peripheral monocytes. Single cell monocytic population was gated by size and complexity. Next, we selected the HLA-DR positive population. Then, three populations were defined based on the expression of CD14 and CD16; Classical Monocytes (CD14hi/CD16neg), Intermediate Monocytes (CD14hi/CD16pos) and Non-Classical Monocytes (CD14lo/CD16pos). We analyzed the level of expression of CD300f and CD300e on the three monocytic populations. (**B**) Analysis of the levels expression of CD300f and CD300e on monocytes from healthy individuals and MS patients. Graphs show the mean fluorescent intensity of CD300f and CD300e expressed on the cell surface of peripheral monocytes from 11 controls and 21 MS patients. Statistically significant differences between groups were determined by a two-tail Mann-Whitney U test: * ≤ 0.05, ** ≤ 0.01.

The lack of CD300f in mice correlates in an experimental model of EAE with increased brain damage^19^. It has been hypothesized that the lower expression of CD300f in monocytes dampens the activation threshold of these cells and promotes their over-reaction once in the central nervous system. Thus, it is conceivable that, through different mechanisms, a decrease in the expression of CD300f on human monocytes and/or other myeloid cells, such as microglia, could exacerbate MS disease. To test this hypothesis, the expression levels of this receptor in different subtypes of peripheral blood monocytes from healthy individuals and MS patients were analyzed by flow cytometry. As shown in Fig. 7A, three populations of peripheral monocytes were evaluated simultaneously: classical monocytes (CD14hi/CD16neg), intermediate monocytes (CD14hi/CD16pos) and non-classical monocytes (CD14lo/CD16pos). The CD300f expression levels on the surface of both, classical and intermediate monocytes (Fig. 7B), were significantly lower in the MS group compared to healthy individuals. No differences were observed within the non-classical population of monocytes. It is noteworthy that CD300e expression was similar for the three monocyte subsets analyzed when comparing healthy and disease populations.

## Discussion

We have previously described the capability of CD300 receptors to associate between them extracellularly forming homo- and hetero-signaling complexes. Moreover, the integration of CD300 molecules in complexes modifies the individual signaling properties of each receptor producing an integrated signal depending on the complex composition^4^. We proposed that the binding among CD300 receptors Ig domains relied on the amino acidic residues, discarding the hypothesis that post-translational modifications such as N- and/or O-glycosylation could participate in CD300 complex formation. In addition to the pair of cysteine residues describing the Ig-V-type fold, CD300 family members display a second pair of Cys amino acids that form a second disulfide bridge within a loop defining the bottom of a solvent-exposed cavity suitable for protein-protein interactions^26^. Our initial hypothesis was that this CD300-specific solvent-exposed cavity would participate in the formation of CD300 complexes. Surprisingly, the association of CD300b with itself and with other CD300 receptors was not affected by eliminating the Cys residues responsible for the second disulfide bridge or by mutations in key residues (tryptophan 55, asparagine112, glutamic acid 63 and tryptophan 103) disturbing the hydrophobic properties and negative electrostatic potential of the cavity^26^.

Our initial goal in this work was to identify the residues on the Ig domain of CD300 molecules responsible for the formation of both homo- and heterocomplexes. As these complexes modulate the overall function of CD300 receptors, we suggested that once identified, SNPs and mutations affecting those residues in human CD300 proteins could participate in the pathophysiology of diseases related to CD300 dysfunction described in murine models. The use of the 3DLigandSite tool allowed us to define some regions on the extracellular domain of CD300b as potential binding sites. 3DLigandSite utilizes protein-structure prediction to provide structural models for proteins that have not been solved, as in the case of CD300b. Ligands bound to structures similar to the query are superimposed onto the model and used to predict the binding site^27^. Five binding clusters were obtained as a result of our query, using the sequence of human CD300b IgG domain.

The data generated by mutating residues defining clusters 2, 3, 4 and 5 seem somehow confusing. First of all, mutation of residues glutamic acid 28 and glutamine 29 in cluster 5 is able to disrupt the formation of complexes of CD300b with itself and with other family members, whereas the disruption of cluster 3 did not change the binding capability of CD300b. The fact that the binding between CD300 wt and CD300b_E28-Q29_ proteins is higher than that between the two mutated proteins (Fig. 2B) strongly suggests the existence of a binding site 1, disrupted by mutations E28A and Q29G, interacting with binding site 2. To prove this hypothesis, we disrupted clusters 2 and 4 by substituting key binding residues asparagine 81 and aspartic acid 84, assuming that cluster 2/4 could constitute binding site 2. From the data shown in Fig. 4 we could extract two conclusions. First, the substitution of both residues within CD300b receptor does not affect complex formation, ruling out cluster 3/4 as the putative binding site 2. Second, as far as mutation of both residues reconstituted the capability of this construct to interact with CD300b_E28A-Q29G_, residues glutamic acid 28 and glutamine 29 are not directly involved in the binding with other residues. Our hypothesis is that substitution of those residues produces a conformational change on the tertiary structure of CD300b Ig domain that blocks the interaction. This hypothesis is reinforced by the analysis of the Arg33Gln mutation found in CD300f in a MS patient (Fig. 5A). In that case, despite the high homology between the Ig domains of CD300b and CD300f (Fig. 5B), the change of arginine to glutamine does not affect the capability of CD300f to form complexes (Fig. 5C). Indeed, the change of residue arginine 33 to a glutamine found in the mutated form of CD300f makes these two amino acids identical to those found at the same position in CD300b (Fig. 5B). The substitution of residues glutamic acid 32 and arginine 33 in CD300f to alanine did not affect the capability of CD300f to form complexes, although in the case of the mutated R33Q form found in the MS patient, it seems that it could affect the stability of the protein (Fig. 6C).

As residues glutamic acid 32 and arginine 33 in CD300f form part of a beta sheet turn, as described by Marquez *et al*.^26^, the homologous residues glutamic acid 28 - glutamine 29 found in CD300b are most probably forming part of a beta sheet turn also (Fig. 5B). Beta sheet turns play a critical role in the folding of some proteins by enabling or allowing interactions between regular secondary structure elements. Mutagenesis studies have shown that changes in the residues within the turns result in variations in protein stability^28^. In order to analyze the effect of substituting arginine 33 in CD300f by a glutamine residue, we used the CUPSAT tool (http://cupsat.tu-bs.de/), which predicts changes in protein stability following point mutations. The prediction model uses amino acid atom potentials and torsion angle distribution to assess the amino acid environment of the mutation site. Additionally, the prediction model can distinguish the amino acid environment using its solvent accessibility and secondary structure specificity^29^. While the substitution of arginine by a glutamine in CD300f does not predict a global effect on the overall protein stability, it produces changes in torsion angles (φ, ψ) that could result in small modifications in the secondary structure. The differences between CD300f and CD300b sequences could explain why changes in similar residues in two proteins with high homology could result in one case in a small change in the secondary structure affecting its binding capability (CD300b), whereas in the case of CD300f seems to affect protein stability independently of its capability to form homocomplexes. Taken together, our data further confirm what has been described by a good number of researchers; there is tremendous variability in the importance of individual amino acids in protein sequences. At sites that are key determinants of stability or activity, even residue substitutions that are generally considered to be conservative can display severe phenotypic effects.

We have shown previously that CD300 complex formation could modulate the function of individual CD300 receptors. By transfecting RBL-2H3 CD300-overexpressing cells with luciferase reporter systems, we found that the combination of CD300 receptors in a complex differentially modulates the signaling outcome^4^. In the case of CD300f, this has been described to be able to deliver both inhibitory^7,15,30^ and activating^31,32^ signals. We have recently proved that in mice, CD300f is able to act as an activating receptor through the formation of complexes with IL4R receptor^21^. By extension, it is conceivable that the function of other CD300 members, as is the case of CD300b, could be regulated similarly. For instance, in Fig. 3 we show that the signaling capability of CD300b is 4-fold enhanced when CD300b_E28A-Q29G_ is triggered in transfected RBL-2H3 cells. These data reinforce our previous results and suggests that in normal conditions, CD300b activating function could be regulated by the formation of homocomplexes and/or heterocomplexes.

MS is a chronic inflammatory of disease that affects the central nervous system (CNS). It is considered an autoimmune disorder initiated by auto-reactive lymphocytes that promote abnormal responses against CNS autoantigens, the nature of which remains elusive. The disease is characterized by the destruction of myelin, a lipidic-based sheath around most nerve fibers acting as an insulator^33^. Immune cell infiltration, mostly composed of monocytes and T and B lymphocytes moving across the blood-brain-barrier promotes inflammation, demyelination, gliosis and neuroaxonal degeneration. MS development has been associated with genetic predisposition, although diverse environmental and stochastic factors could trigger the disease and/or influence its penetrance^33^. A neuroprotective role for CD300f was reported some years ago in a murine EAE model of MS^19^. In the mouse model that was deficient for CD300f, an increase of nitric oxide and pro-inflammatory cytokines were detected, as was the enhancement of demyelination and worsened clinical scores. The authors concluded that CD300f behaved as a negative regulator of myeloid effector infiltrated cells in autoimmune demyelination^19^. Moreover, the ligands for CD300f are enriched in CNS white matter^24^ and peripheral nerves^20^, both of which are rich in myelin. In the current work, we have described a unique mutation in an MS patient (rs141171369) located in exon 2 of CD300f gene. Although that mutation seems to affect CD300f stability in COS7 transfected cells, we did not demonstrate R33Q to be responsible for the significant reduction in the expression of CD300f on the patient’s monocyte populations. More interestingly, we found that levels of CD300f were significantly diminished on the cell surface of monocytes from MS patients compared to healthy individuals (Fig. 7). These results suggest that infiltrating human monocytes with diminished levels of CD300f in MS patients could be more active and thus more prone to promote inflammation paralleling that which has been described for the CD300f KO mice.

## Methods

### Cells and antibodies

COS-7 and RBL-2H3 cells were grown in DMEM containing 10% heat inactivated FBS, 2mM L-glutamine, 1 mM sodium pyruvate, 100IU/mL penicillin and 100 µg/mL streptomycin. P815 cells were maintained in RPMI 1640/L-glutamine medium supplemented with 10% heat inactivated FBS, 25 mM HEPES, 2mM L-glutamine, 100 IU/mL of penicillin, and 100 μg/mL of streptomycin. Human peripheral blood mononuclear cells (PBMC) were isolated by Ficoll-Isopaque density gradient centrifugation (Gibco-Thermofisher Scientific, Waltham, MA, USA) and stored in liquid nitrogen until use. Anti-Hemagglutinin (HA).11 mAb ascites was from Covance (Princeton, NJ, USA). Anti-FLAG M2^®^ mAb peroxidase-conjugated, anti-DNP IgE and F(ab’)_2_ sheep anti-mouse IgG were from Sigma-Aldrich, St. Louis, MO, USA. Anti-Rat High affinity IgE Receptor mAb was obtained from BD Biosciences, Franklin Lakes, NJ, USA. Anti-HA12CA5 mAb and anti-Myc9E10 mAb were previously described^4^. Streptavidin-HRP was purchased from Roche, Basel, Switzerland. Goat anti-mouse Ig-HRP was from Santa Cruz Biotechnology, Dallas, TX, USA. Anti-human CD300f UPD2 mAb was described before^7^. FITC-conjugated anti-mouse rabbit polyclonal antibody was from DAKO-Agilent Technologies, Santa Clara, CA, USA. PE anti-human CD300f was from Milteny Biotec, Bergisch Gladbach, Germany and PE anti-human CD300e from Abcam, Cambridge, UK. PerCP/Cy5.5 anti-human HLA-DR was purchased from Biolegend, San Diego, CA, USA and pacific blue anti-human CD14 and APC anti-human CD16 from Immunostep, Salamanca, Spain.

### DNA Reagents

Mammalian Flag-CD300b, HA-CD300a, HA-CD300b, HA-CD300c, HA-CD300c, HA-CD300e, HA-CD300f constructs were described previously^4^. Flag CD300b_R95G-Q97G_, Flag CD300b_E28A-Q29G_, Flag-CD300b_N81A-D84A_ and HA-CD300f_R33Q_ constructs were generated with mutagenic oligonucleotides according to the instructions of the GeneArt site-directed mutagenesis system kit (Thermofisher) and further confirmed by DNA sequencing under Big Dye^TM^ cycling conditions on an Applied Biosystems 3730xl DNA Analyzer (Macrogen Inc,, Seoul, South Korea). Details have been summarized in Supplementary Table 1.

### Cell transfections

COS-7 cells (6 × 10^5^) were transiently transfected with LyoVec^TM^ Reagent (InvivoGen, San Diego, CA, USA) according to the manufacturer’s instructions. Analysis of protein stability in COS-7 transfected cells was carried out by cycloheximide chase assay (10 µM) at the indicated times. For the generation of RBL-2H3 stable transfectants, 20 × 10^6^ cells were electroporated in the presence of 20 µg of the appropriate linearized construct at 250 V, 960µF and 100Ω in a Gene Pulser Electroporator (Bio-Rad, Hercules, CA, USA). Transfectants were selected and maintained in culture with the appropriate selection antibiotic. G418 (BioWhittaker-Lonza, Walkersville, MD, USA) and/or puromycin (Sigma-Aldrich) were used at 1 mg/mL and 1 µg/mL respectively. Positive cells were further selected by immunostaining with the appropriate antibodies and sorting with magnetic Dynabeads^®^ M-450 coated with Sheep anti-Mouse IgG (Dynal-Thermofisher).

### Flow cytometry

Cell surface expression of the desired molecules was tested by direct or indirect immunofluorescence following standard techniques^32^. Analysis was performed using a FACSAria cell sorter (Becton Dickinson) instrument and Flow Jo software (Tree Star Inc., Ashland, OR, USA).

### Immunoprecipitation and western blot analysis

Cells were lysed at 4 °C for 20 min using 1% Triton X-100 (Tx-100)-containing buffer described previously^3^. Cell lysates were clarified by centrifugation at 16,000 × g for 15 min at 4 °C. Crude lysates were pre-cleared for 1 h at 4 °C using 20 µL of IgG Sepharose 6 Fast Flow (GE Healthcare Life Sciences, Chicago, IL, USA). Two additional pre-clearings were conducted for 30 min at 4 °C. For immunoprecipitations, pre-cleared lysates were incubated with 30 µL of Protein G-Sepharose beads (GE Healthcare) and 1 µg of Ab for 3 h at 4 °C. Proteins in the crude lysates (2%) and immunoprecipitates were separated by SDS-PAGE and transferred onto polyvinylidene difluoride (PVDF) filters (Millipore, Billerica, MA, USA). Filters were blocked with 5% skim-milk and then probed with the indicated Abs at appropriate dilutions. Bound Abs were detected using West Pico Supersignal kit (Pierce-Thermofisher). Densitometric quantification was performed by using ImageJ software.

### Luciferase assays

RBL-2H3 transfectants were transiently electroporated with a luciferase reporter plasmid (pT81Luc) containing three tandem copies of the distal NFAT/AP-1 site of the murine IL-2 promoter^3^ (0.5 µg/10^6^ cells) and a TK Renilla construct (Promega, Madison, WI, USA) (0.1 µg/10^6^ cells). Twenty-four hours post transfection, 1.5 × 10^6^ cells were stimulated for 7 h with the indicated antibodies using the murine mastocytoma P815 cell line as the presenting cell (1 × 10^6^). Plastic-coated anti-DNP IgE (5 µg/mL) was used as positive control for RBL-2H3 cells stimulation. The P815 cell line cultured in supplemented RPMI 1640/L-glutamine medium alone was used as a negative control. Post-nuclear lysates were obtained as described^3^ and luciferase activity was measured according to the Dual Luciferase Report kit manual (Promega) using a FB12 Luminometer (Berthold, Bad Wildbad Germany).

### CD300f and CD300b sequencing of exon 2 and mRNA

Genomic DNA from peripheral blood samples was obtained using standard methods. Primers (Supplementary Table 1) were designed to amplify exon 2 of genes Cd300f and Cd300b genes encoding for the immunoglobulin domain. PCR amplifications were performed in a final volume of 50 µL. Each reaction consisted of 0.4 µmol/L final concentration of each primer, 0.2 mmol/L of each dNTP (Promega), 2 mmol/L of MgCl2, 2.5 units of GoTaq DNA Polymerase (Promega), and 50 ng of template genomic DNA. Amplifications were carried out in a PTC 100 Thermal Cycler (MJ Research, Cambridge, MA) using an initial denaturation step at 95 °C for 2 min, followed by 30 cycles of 95 °C for 1 min, 65 °C for 30 sec and 72 °C for 1 min, and a final elongation step at 72 °C for 10 min. Reactions were run in 1.5% agarose gels with Tris-borate-EDTA (TBE) buffer for 3 h to verify the presence of single and clear bands. PCR products were digested with EXO-SAP-IT (Thermofisher) following the manufacturer’s instructions and sequenced under Big Dye^TM^ cycling conditions as described before. RNA isolated from PBMC from LF6105 patient using Purelink RNA micro kit following manufacturer’ s instructions (Pierce-Thermofisher) was retrotranscribed to cDNA using High-Capacity cDNA Reverse Transcription Kit (Applied Biosystems, Foster City, CA, USA) and full length cDNA was amplified using specific primers (Supplementary Table 1). PCR products were cloned into the pSpark cloning vector following the kit instructions (Canvax, Cordoba, Spain) and sequenced as described.

### Patients with MS

All the PBMC samples included in the study were collected from MS patients who were not receiving immunomodulatory or immunosuppressive treatment at the time of blood drawing. For the flow cytometry studies, PBMC from 21 relapse-onset MS patients [52.3% females; mean age (standard deviation, SD): 33.9 years (12.0); mean number of relapses in the previous 2 years: 1.4 (0.6); mean disease duration: 7.1 years (9.0); median EDSS at the time of blood collection (interquartile range): 1.5 (1.0–2.3)] and 11 healthy controls [54.5% females; mean age: 31.3 years (4.2)] were included in the study. For the sequencing study, DNA samples from 50 relapse-onset MS patients [44.0% females; mean age: 28.1 years (10.7); mean number of relapses in the previous 2 years: 2.6 (1.1); mean disease duration: 9.7 years (14.8); median EDSS at the time of blood collection (interquartile range): 2.0 (1.5–3.0)] were included in the study. Informed consent for genetic analysis was provided by all patients. All experimental methods were approved by the Hospital Universitari Vall d’Hebron Ethics Committee and carried out in accordance with the guidelines and regulations.

## Electronic supplementary material

Table 1
