# Supplementary material for: Effect of Specific Mutations in Cd300 Complexes Formation; Potential Implication of Cd300f in Multiple Sclerosis
Source: Sci Rep. 2017 Oct 19;7:13544. doi: 10.1038/s41598-017-12881-8 (PMC5648872; doi:10.1038/s41598-017-12881-8)
Supplement: Supplementary file 1 — Table 1 [file 41598_2017_12881_MOESM1_ESM.doc]

**SUPPLEMENTARY TABLE**

| **NAME** | **FORWARD**  **REVERSE** | **SEQUENCE** |
| --- | --- | --- |
| hCD300b E28A-Q29G | Forward | GTGAGAGCCCCAG**C**G**GG**GGGGTCCCTGACG |
| hCD300b E28A-Q29G | Reverse | CGTCAGGGACCCC**CC**C**G**CTGGGGCTCTCAC |
| hCD300b R95G-N97A | Forward | ATGGAGGGGCTC**G**GGCGAG**C**TGACGCAGAT |
| hCD300b R95G-N97A | Reverse | ATCTGCGTCA**G**CTCGCC**C**GAGCCCCTCCAT |
| hCD300b N81A-D84A | Forward | CCATCAAGGAC**GC**TCAGAAAG**C**CCGCACGTTCAC |
| hCD300b N81A-D84A | Reverse | GTGAACGTGCGG**G**CTTTCTGA**GC**GTCCTTGATGG |
| hCD300f E32A-R33G | Forward | GTGAATGGCCTGG**C**G**G**GGGGCTCCTTGACC |
| hCD300f E32A-R33G | Reverse | GGTCAAGGAGCCCC**C**C**G**CCAGGCCATTCAC |
| hCD300f R33Q | Forward | TGAATGGCCTGGAGC**A**GGGCTCCTTGACCG |
| hCD300f R33Q | Reverse | CGGTCAAGGAGCCC**T**GCTCCAGGCCATTCA |
| hCD300b Exon 2 | Forward | ctcaggcaacaggagctgta |
| hCD300b Exon 2 | Reverse | gggagcaggtaagagcactg |
| hCD300f Exon 2 | Forward | GTTTTGTCTGCGGAAGGTGT |
| hCD300f Exon 2 | Reverse | GGACCTCAGAGACCAGGACA |
| hCD300f cDNA | Forward | AACGGGGACCTGTCTGAAG |
| hCD300f cDNA | Reverse | GTCGATGAGGCAGGAGTGTGCTCACAG |

Table 1: Oligos used in this study. Nucleotide changes introduced in the sequence for amino acid substitution are shown in bold letters.
